# Use of enzymatic tools for biomonitoring inorganic pollution in aquatic sediments: a case study (Bor, Serbia)

**DOI:** 10.1186/1752-153X-7-59

**Published:** 2013-03-28

**Authors:** Marioara Nicoleta Filimon, Dragos V Nica, Vasile Ostafe, Despina-Maria Bordean, Aurica Breica Borozan, Daliborca Cristina Vlad, Roxana Popescu

**Affiliations:** 1 Department Biology-Chemistry, West University of Timisoara, Faculty Chemistry-Biology-Geography, Pestalozzi 16, Timisoara, RO 300315, Romania; 2 West University of Timisoara, Multidisciplinary Research Platform “Nicholas Georgescu – Roengen”, Advanced Environmental Research Laboratories, Oituz 4, Timisoara, 300086, Romania; 3 Faculty of Animal Sciences and Biotechnologies, Banat’s University of Agricultural Sciences and Veterinary Medicine from Timisoara, Calea Aradului 119, Timisoara, RO 300645, Romania; 4 Faculty of Food Processing Technology, Banat’s University of Agricultural Sciences and Veterinary Medicine from Timisoara, Calea Aradului 119, Timisoara, RO 300645, Romania; 5 Faculty of Agriculture, Banat’s University of Agricultural Sciences and Veterinary Medicine from Timisoara, Calea Aradului 119, Timisoara, RO 300645, Romania; 6 Department of Pharmacology and Biochemistry, University of Medicine and Pharmacy “Victor Babes”, 300041, E. Murgu, 2, Timisoara, Romania; 7 Department of Cellular and Molecular Biology, University of Medicine and Pharmacy “Victor Babes”, 300041, E. Murgu, 2, Timisoara, Romania

**Keywords:** Sediment bacterial community, Inorganic pollution, Enzymatic indicator of sediment quality, Environmental biomonitoring

## Abstract

**Background:**

Sediment bacterial communities are key players in biogeochemical cycling of elements in the aquatic environment. Copper mining, smelting, and processing operations located in Bor area (Serbia) are major environmental hot spots in the lower Danube Basin and Western Balkans. In the present study, we evaluate the influence of trace element (TE) concentration in sediments and physico-chemical properties of water on sediment microbial communities in water streams adjacent to the Copper Smelter Complex Bor (RTB Bor, Serbia). The degree to which metabolic activities of bacterial biota inhabiting differently polluted sites is inhibited by inorganic pollution were compared using selected enzymatic bioindicators.

**Results:**

Cu, Zn, Pb, and As concentrations systematically exceeded the target values for metal loadings in aquatic sediments. Water electrical conductivity (WEC) followed the same pattern of spatial variation, irrespective of season. Interestingly, the most intense enzymatic activity occurred at the reference site although this site showed the greatest TE levels in aquatic sediments. Catalase activity (CA), potential dehydrogenase activity (PDA), actual dehydrogenase activity (ADA), urease activity (UA), and phosphatase activity (PA) in aquatic sediments displayed heterogeneous patterns of spatio-temporal variation. Inorganic pollution greatly affected CA, ADA, and PDA, but much less so UA and PA. Canonical correlation analysis showed that pH and WEC were the strongest determinants of enzymatic activity in bacterial biota, with the latter variable being reversely correlated with the enzymatic indicator of sediment quality (EISQ). The median values of EISQ increased with distance from the major sources of pollution. In addition, it was found that sites with different degrees of inorganic pollution can be appropriately classified by applying cluster analysis to EISQ, TE levels in sediments, and physico-chemical properties of water.

**Conclusions:**

Because EISQ can precisely identify changes in overall enzymatic activity of sediment bacterial communities, this enzymatic bioindicator has a great potential for biomonitoring the current status of inorganic pollution in aquatic ecosystems.

## Background

River sediments play a key role in biogeochemical cycling of elements in the aquatic environment [1]. Sediments are either suspended in the water column, i.e., suspended sediments, or deposited on the river beds, i.e., bedded sediments [2]. Trace elements (TE) from anthropic sources enter the water column, are absorbed onto sediment particles and then settle on the river/stream beds [3]. The degree of pollutant distribution, accumulation and mobility in sediments is influenced by various factors, such as sediment texture, mineralogical composition, absorption and desorption processes, or oxidation-reduction equilibrium [4]. Once deposited in sediments, pollutants interfere in trophic chains via bioaccumulation and biomagnification [5]. In addition, sediment deposition has negative effects on biota and physical habitat, which occasionally result in limitation of photosynthesis, changes in population structure, or spawning habitat degradation [1]; therefore, measurement of TE concentration in sediments functions as an important tool for assessing contamination in aquatic environments [6].

Although such geochimical criteria provide a measure of contaminant retention in aquatic sediments, they do not provide objective information concerning their impact on biotic components of an ecosystem. Because microbial communities are deeply involved in metal mobility [7], several study authors have investigated the relationships between TE concentration and microbial enzymatic activity to assess the degree of pollution in aquatic sediments. It was found that each bacterial community reacts differently in response to environmental pollution. For example, in the same environments microbial activity (e.g., dehidrogenase activity) was inhibited by trace metals [8-10], whereas in other bacterial communities the microbial activity was insensitive even to high metal loadings in soil/sediments [10,11]. In sum, specialized biomarkers are needed when assessing the impact of anthropic-induced pollution on microbial enzymatic activity. If the study is oriented toward practical applications, i.e., in the present case, the investigations should be focused on finding whether pollutants have negative effects on those microbial activities that are thought to be ecologically important [12]. Various enzymatic activities, such as catalase activity (CA), potential dehydrogenase activity (PDA), actual dehydrogenase activity (ADA), urease activity (UA), or phosphatase activity (PA), were shown to be related to the degree of inorganic contamination in soils and sediments [13-15]. Measurement of these enzymatic activities may therefore allow environmental scientists to assess the impact of inorganic pollution on soil/sediment bacterial biota. This approach provides the basic evidence needed to assume the occurrence of contamination with xenobiotics in natural environments without predicting the metabolic response at organism level at the given level of exposure [16]. The enzymatic indicator of sediment/soil quality (EISQ), by contrast, provides a more comprehensive overview of the changes that occur in microbial metabolism in response to environmental pollution by simultaneously assessing the additive effect of inorganic contaminants on several key enzymatic activities such as CA, PDA, ADA, UA, and PA [17,18]. EISQ was shown to decrease in a dose-dependent manner in response to zinc, cadmium, or lead exposure under laboratory conditions [15,19]. In natural environments, EISQ has proved to be strongly correlated with levels of chlorides, sulfates, magnesium, natrium, iron, lead, copper, and zinc in aquatic sediments [20]. This bioindicator may hence serve as a potential solution to the aforementioned impediment.

**Figure 1:**
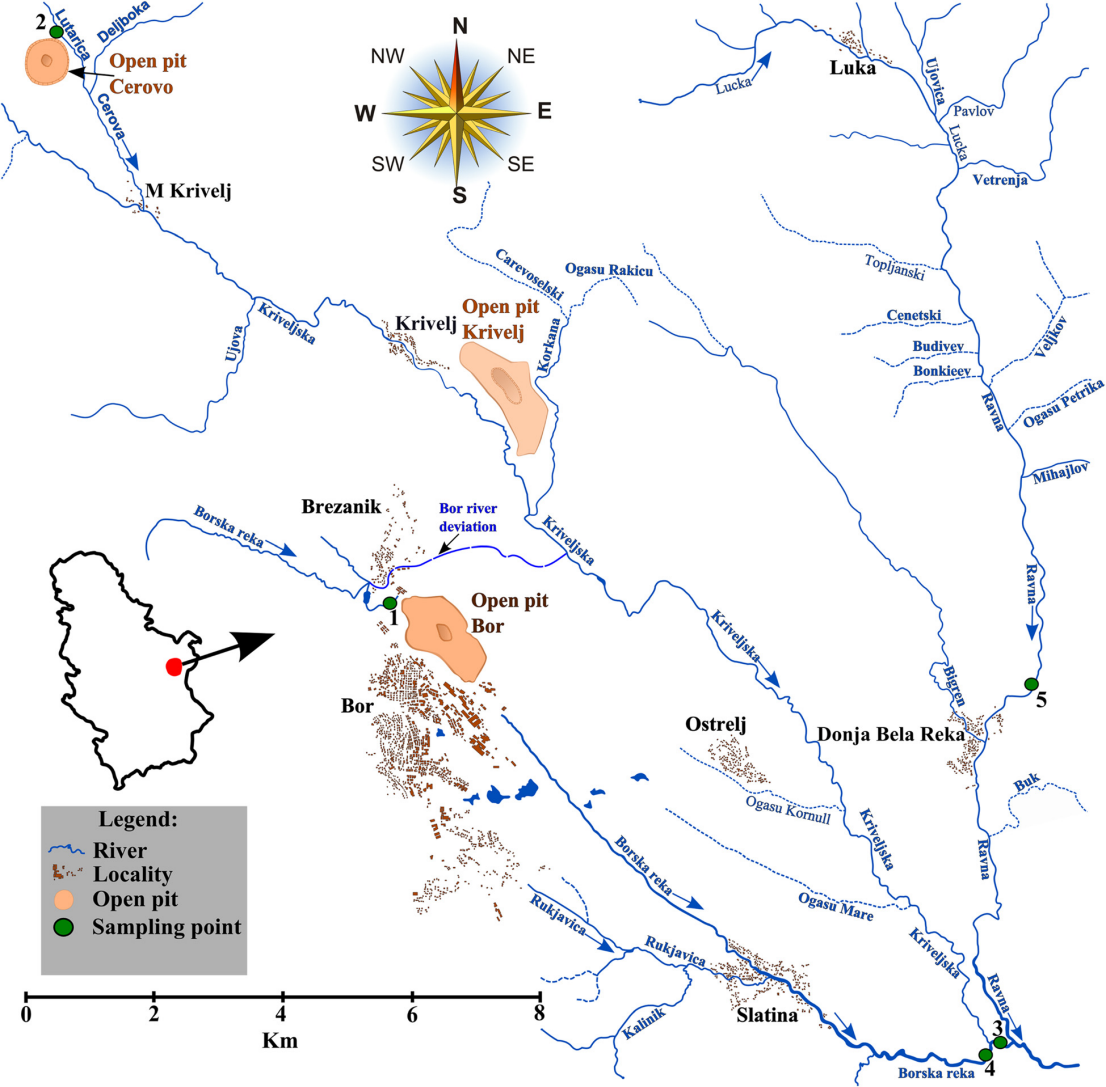
**Map showing the location of sampling sites.***Legend.* 1, site S1; 2, site S2; 3, site S3; 4, site S4; 5, site SR.

The Danube River flows for 2,872 km through Central and Eastern Europe before pouring into the Black Sea via the Danube Delta [21]. The Timok River is one of the most polluted water streams in Serbia, and an important right tributary within the lower Danube River Basin [22]. The Bor River (syn. Borska Reka River) flows through the region of Bor, which is well known for the extensive soil, water, and air pollution with inorganic compounds caused by non-ferrous metal mining, smelting, and processing operations located in this area [23], and finally flows into the Timok River [24]. The main tributary of the Bor River are the Kriveljska and Ravna rivers (Figure 1). The Ravna River is located relatively far from the major sources of current pollution within the Bor area, such as the Cerovo, Bor and Krivelj open pit mines (Figure 1). By contrast, the Kriveljska River collects the highly polluted waters of the Cerova River [25] and passes next to the Bor open pit mine (Figure 1). A tailing dam, i.e., the Veliki Krivelj tailing dam, was constructed in its valley in 1982 by deviating the river (tunnel and collector) and damming the river downstream and upstream, and then further extended in 1990 [26]. Together with additional sources of polluted waters, such as waste wasters from the “Jama Bor” underground mine or other types of industrial waters, it is estimated that almost 1,285 tons of iron (Fe), 502 tons of copper (Cu), 1.5 tons of nickel (Ni), 0.5 tons of arsenic (As), 52 tons of zinc (Zn), 2 tons of lead (Pb), 0.3 tons of cadmium (Cd), and 6 tons of manganese (Mn) are discharged every year, without any treatment, in the hydrographic basin of the Bor River [26]. These pollutants finally enter the Danube River via the Timok River, and therefore it is easy to anticipate the potential risks that pollution in Bor area can pose to aquatic ecosystem from the lower Danube Basin.

The Bor River is actually considered to be one of the most polluted water streams in Europe [24]. Despite containing high levels of heavy metals and having a low pH, this river accommodates bacterial life [27]. However, no field survey has investigated the effects of extensive inorganic pollution on enzymatic activity of sediment bacterial communities. This type of pollution is generally associated with changes in physico-chemical properties of the water and enrichment of aquatic sediments in heavy metals [1]. The levels of copper (LCu), lead (LPb), zinc (LZn), nickel (LNi), chromium (LCr), arsenic (LAs), and iron (LFe) in aquatic sediments were chosen in the present study because these TEs have been shown to frequently occur in wastewaters resulting from local mining and metal processing activities, which are routinely discharged within the Bor River Basin [26]. Among the physico-chemical properties of water, the amount of dissolved oxygen in water (DO), activity of the solvated hydrogen ion (pH) and water electrical conductivity (WEC) are important when examining the quality of aquatic habitats [1,22,24]. Consequently, our purpose in this paper was to address the following practical questions concerning the problems of using microbial enzymatic activity as biomarker for biomonitoring inorganic contamination in Bor area:

1. What is the level to which various trace elements accumulated in water stream beds around the city of Bor at key sampling points, and which is the current impact of inorganic pollution on water quality in these rivers?

2. How are catalase activity (CA), potential dehydrogenase activity (PDA), actual dehydrogenase activity (ADA), urease activity (UA), or phosphatase activity (UA) of sediment bacterial biota influenced by TE deposition in water stream sediments and the physico-chemical properties of the water?

3. Can EISQ serve as a reliable biomarker of inorganic pollution in Bor area, what are the limitations associated therewith, and how can such impediments be overcome?

## Results and discussions

### Physico-chemical properties of water

The physico-chemical properties of water are given in Table 1. For each location and season, DO, WEC and pH are expressed as median values with standard deviation. Among physico-chemical properties of water, WEC and pH were heteroscedastic (p < 0.05), whereas DO satisfied the preconditions of homoscedasticity (p > 0.05). It was found that the measured values were significantly different among sampling sites (p < 0.05). WEC correlated positively with pH, but negative relationships were reported between either pH or WEC and DO (Table 2).

**Table 1:** Physico-chemical properties of water at investigated sites

**Sample**	**DO**	**pH**	**WEC**	**Color**	**Odor**
1.1.	5.091 ± 0.567	2.357 ± 0.321	7290 ± 4193	light grey	no odor
1.2.	5.174 ± 1.289	3.794 ± 0.721	908 ± 261	no color	no odor
1.3.	7.987 ± 0.361	3.791 ± 0.227	1995 ± 1023	light grey	“rotten egg” odor
1.4.	7.059 ± 1.361	5.123 ± 0.225	1832 ± 607	blackish	“rotten egg” odor
1.5.	8.970 ± 1.741	7.765 ± 0.761	485 ± 231	no color	no odor
2.1.	5.751 ± 1.082	2.458 ± 0.445	7450 ± 3098	light grey	no odor
2.2.	5.832 ± 0.602	3.934 ± 0.631	927 ± 422	no color	no odor
2.3.	8.081 ± 2.321	3.981 ± 0.429	1879 ± 1205	medium grey	“rotten egg” odor
2.4.	7.674 ± 0.972	5.352 ± 0.895	1913 ± 887	blackish	“rotten egg” odor
2.5.	9.134 ± 0.341	7.816 ± 0.806	495 ± 334	no color	no odor
3.1.	5.876 ± 0.965	2.211 ± 0.274	7050 ± 4031	light grey	no odor
3.2.	6.093 ± 1.772	8.070 ± 0.552	911 ± 553	no color	no odor
3.3.	8.979 ± 0.871	4.311 ± 0.612	1771 ± 1028	light grey	“rotten egg” odor
3.4.	7.821 ± 1.429	5.639 ± 0.186	1943 ± 881	blackish	“rotten egg” odor
3.5.	8.952 ± 1.602	7.989 ± 0.388	497 ± 261	no color	no odor
4.1.	5.368 ± 0.769	2.301 ± 0.306	7290 ± 2010	light grey	no odor
4.2.	5.833 ± 1.003	7.940 ± 0.621	908 ± 712	no color	no odor
4.3.	8.743 ± 2.412	3.963 ± 0.481	1995 ± 1054	medium grey	“rotten egg” odor
4.4.	7.502 ± 2.971	5.263 ± 0.551	1832 ± 723	blackish	“rotten egg” odor
4.5.	8.612 ± 1.231	7.942 ± 0.428	485 ± 210	no color	no odor

*Abbreviations.* DO, dissolved oxygen in water; pH, activity of the (solvated) hydrogen ions in water; WEC, electrical conductivity of water; 1.1., site S1 (July 2011); 1.2., site S2 (July 2011); 1.3., site S3 (July 2011); 1.4., site S4 (July 2011); 1.5., site S5 (July 2011); 2.1., site S1 (November 2011); 2.2., site S2 (November 2011); 2.3., site S3 (November 2011); 2.4., site S4 (November 2011); 2.5., site S5 (November 2011); 3.1., site S1 (March 2012); 3.2., site S2 (March 2012); 3.3., site S3 (March 2012); 3.4., site S4 (March 2012); 3.5., site S5 (March 2012); 4.1., site S1 (July 2012); 4.2., site S2 (July 2012); 4.3., site S3 (July 2012); 4.4., site S4 (July 2012); 4.5., site S5 (July 2012).

**Table 2:** Correlations between enzymatic and abiotic variables within the area of interest

	**ADA**	**PDA**	**UA**	**PA**	**CA**	**DO**	**pH**	**WECs**	**LCu**	**LPb**	**LZn**	**LNi**	**LCr**	**LAs**	**LFe**
**ADA**		**0.797**	-0.077	-0.151	**0.519**	0.288	**0.451**	-0.413	0.378	0.219	0.204	0.084	0.374	0.177	0.079
**PDA**			0.026	0.130	**0.460**	0.283	0.293	-0.442	0.225	0.107	0.100	0.043	0.172	0.097	**0.175**
**UA**				0.313	-0.308	0.000	0.202	-0.117	0.017	-0.241	0.039	-0.016	0.062	0.110	-0.015
**PA**					-0.056	0.122	0.081	-0.011	0.218	0.245	0.266	0.347	0.129	0.326	0.477
**CA**						0.242	**0.484**	-0.444	0.209	0.269	0.162	0.016	0.144	0.038	0.203
**DO**							**0.535**	**-0.479**	**0.463**	0.342	**0.568**	**0.603**	0.391	**0.534**	0.195
**pH**								**-0.774**	**0.580**	0.313	**0.603**	**0.474**	**0.558**	**0.481**	0.245
**WEC**									**-0.471**	-0.286	**-0.481**	**-0.475**	-0.336	-0.381	-0.281
**LCu**										**0.851**	**0.859**	**0.665**	**0.873**	**0.911**	**0.621**
**LPb**											**0.689**	**0.466**	**0.627**	**0.829**	**0.785**
**LZn**												**0.843**	**0.830**	**0.817**	**0.507**
**LNi**													**0.611**	**0.621**	0.230
**LCr**														**0.771**	0.378
**LAs**															**0.704**
**LFe**															

*Note.* Figures in bold type defines values significant correlations (p < 0.05).

*Abbreviations.* ADA, actual dehydrogenase activity in sediment bacterial communities; PDA, potential dehydrogenase activity in sediment bacterial communities; UA, urease activity in sediment bacterial communities; PA, phosphatase activity in sediment bacterial communities; CA, catalase activity in sediment bacterial communities; DO, dissolved oxygen in water; pH, activity of the (solvated) hydrogen ions in water; WEC, electrical conductivity of water; LCu, copper levels in aquatic sediments; LPb, lead levels in aquatic sediments; LZn, zinc levels in aquatic sediments; LNi, nickel levels in aquatic sediments; LCr, chromium levels in aquatic sediments; LAs, arsenic levels in aquatic sediments; LFe, iron levels in aquatic sediments.

Water color and odor varied depending on location, but these features were not influenced by season (Table 1). Water had no color at sites S2 and SR, whereas its color ranged from grey at sites S1 and S3 to blackish at site S4. In addition, water samples from sites S3 and S4 had an unpleasant “rotten egg” odor. These results suggested that elevated levels of trace elements and intense activity of sulfate-reducing bacteria may account for bad water quality at these sampling sites [28,29]. Metal processing in the Copper Smelter Complex Bor (RTB Bor) relies on an outdated processing technology, i.e., classic pyrometallurgical processing, that uses sulfur dioxide gas (SO_2_) to produce H_2_SO_4_ with less than 50% degree of utilization [30]. The Kriveljska and Bor rivers gather the waste waters from the “Jama Bor” underground mine, industrial waters from the city of Bor, drainage waters from the Veliki Krivelj tailing dam, Cerovo, Krivelj, and Bor open pit mines, and overburden waters from the Saraka stream [26,31]. Long-term studies revealed that in the Bor River, downstream of confluence with the Kriveljska River, sulfate concentrations (SO_4_^2-^) in water ranged from 880 to 3,235 mg/L [32]. These levels exceed, by far, the maximal sulfate concentration allowed in drinking water according to the secondary standards of U.S. Environmental Protection Agency (EPA), i.e., 250 mg/L [33]. Therefore, such information may not only explain unusual water color and odor at sites S3 and S4, but they may also justify the local nickname of the Bor River downstream the confluence with the Kriveljska River, which is the Black River (Crna reka) [32].

WEC in freshwater streams should range between 150 and 500 μS/cm (i.e., micro Siemens per cm) to sustain healthy aquatic ecosystems [34]. In the present study, only the measured levels for site SR were shown to vary within this range; these values were the lowest among investigated locations. The highest WEC levels were observed at site S1, whereas the measured values at site S3 were close to those encountered at site S4. Interestingly, WEC followed the same pattern of spatial variation, irrespective of season, and the corresponding values varied within similar range for each site. Because WEC is directly related to the concentration of salts dissolved in water [35], one can conclude that contaminant loading in water remained uniform between July 2011 and July 2012 for all investigated locations. This suggests that wastewater release upstream of all sampling sites was constant throughout the experimental period.

The highest DO levels were systematically detected at site SR, as well as at site S3 in March 2012 and July 2012. In contrast, the lowest DO concentrations were reported for sites S1 and S2. To support fish life the minimum amount of DO must exceed 4–5 mg/L [36], whereas the optimal values range between 7.0 and 11.0 mg/L [37]. Although the measured levels at sites S2, S3, and S4 lied within this range, the Bor River Basin is devoid of fish life [38]. A recent study showed that this river is home to a scarce macrozoobenthos community including larvae, nymphs, and adults of *Diptera* order, but not to members of *Mollusca*, *Isopoda*, *Oligochaeta*, or *Hirudinea* order/class. Althouhg neither macroalgae nor macrophytic vegetation were observed at investigated sites, small communities of *Sphaerotilus* bacteria were reported to exist [27]. Exposure to intensive long-term heavy metal pollution can be held accountable for damaging the native aquatic ecosystems [22,23,26].

### TE levels in aquatic sediments

The observed medians for TE loadings in aquatic sediments and their standard deviations are shown in Table 3, as well as the Dutch and German target values for these TEs. LCu, LPb and LNi were homoscedastic (p > 0.05), but not LZn, LCr, LAs and LFe (p < 0.05). Nonparametric analysis showed that neither LCu, LPb nor LNi were significantly influenced by site location (p > 0.05). TE loadings in aquatic sediments were shown to be positively correlated with each other, excepting LFe which was independent of LZn, LNi, and LCr (Table 2). Several study authors have reported different levels to which TEs accumulated in aquatic sediments within the Danube River Basin [39-41]. However, LCu and LAs greatly exceeded these levels. Similar trends were frequently reported for LZn and LPb. In contrast, LNi and LFe usually varied within the same range. When compared the results with the Dutch or German target values for metal loadings in aquatic sediments, the measured values did frequently exceed these standards, excepting LNi (Table 3).

**Table 3:** Trace element loadings in aquatic sediments

	**LCu**	**LPB**	**LZn**	**LNi**	**LCr**	**LAs**	**LFe**
1.1.	497.012 ± 261.23	45.323 ± 10.241	87.828 ± 42.581	4.629 ± 0.923	34.215 ± 12.903	96.217 ±23.088	23.002 ±16.603
1.2.	1491.629 ± 190.238	125.619 ± 23.298	137.504 ± 54.261	4.695 ± 0.231	13.593 ± 12.094	182.707 ± 76.142	2190.084 ± 51.371
1.3.	1097.572 ± 319.027	160.991 ± 39.903	140.428 ± 72.945	3.272 ± 1.274	10.722 ± 5.894	259.295 ± 172.074	3361.688 ± 193.092
1.4.	1747.989 ± 424.722	64.905 ± 17.072	106.701 ± 43.706	5.424 ± 0.254	15.985 ± 7.439	24.127 ± 12.994	8.584 ± 4.026
1.5.	324.123 ± 281.556	43.123 ± 9.342	222.123 ± 83.293	7.291 ± 1.712	13.907 ± 12.093	13.723 ± 5.096	8.514 ± 3.339
2.1.	363.687 ± 120.502	47.384 ± 12.723	231.192 ± 31.528	7.529 ± 0.925	13.563 ± 2.055	17.693 ± 12.856	9.165 ± 5.015
2.2.	3770.809 ± 501.235	262.922 ± 42.311	2294.104 ± 305.812	11.227 ± 2.157	77.882 ± 54.737	333.726 ± 154.432	3299.040 ± 1266.065
2.3.	4433.846 ± 320.026	400.128 ± 59.637	3241.251 ± 577.243	12.871 ± 2.815	120.305 ± 48.862	523.103 ± 187.963	3519.447 ± 823.497
2.4.	3982.523 ± 448.204	360.164 ± 41.723	2923.382 ± 219.939	11.129 ± 1.997	121.019 ± 51.993	450.725 ± 152.209	4377.842 ± 265.936
2.5.	6283.177 ± 1218.743	480.102 ± 39.271	7150.596 ± 1023.724	17.528 ± 3.063	211.177 ± 79.451	858.593 ± 302.665	7150.245 ± 1088.278
3.1.	515.600 ± 257.692	66.223 ± 13.026	221.754 ± 51.038	4.229 ± 0.416	20.652 ± 13.634	92.754 ± 43.025	2127.441 ± 319.037
3.2.	2851.629 ± 403.271	211.698 ± 51.249	1170.629 ± 286.566	5.203 ± 0.684	40.944 ± 28.784	309.941 ± 152.227	3164.483 ± 1259.025
3.3.	1537.691 ± 284.361	120.872 ± 27.313	1735.728 ± 359.244	14.850 ± 1.274	15.229 ± 8.502	257.579 ± 104.429	34.661 ± 26.904
3.4.	2142.184 ± 430.009	81.553 ± 9.099	1470.534 ± 477.209	4.772 ± 0.619	113.380 ± 73.959	223.329 ± 138.862	34.220 ± 9.266
3.5.	4348.423 ± 385.206	115.426 ± 17.272	7190.323 ±1301.358	16.819 ± 3.219	125.506 ± 174.097	721.801 ± 302.940	42.688 ± 27.055
4.1.	501.724 ± 131.049	56.177 ± 16.055	152.718 ± 72.905	5.447 ± 1.441	23.619 ± 13.056	32.743 ± 21.623	7.170 ± 1.426
4.2.	2585.617 ±494.654	151.628 ± 25.014	2170.356 ± 567.023	7.202 ± 1.723	54.928 ± 24.174	219.506 ± 132.038	2749.279 ± 173.032
4.3.	3237.605 ± 1020.392	220.827 ± 6.251	1873.529 ± 510.698	8.896 ± 1.529	103.205 ± 75.022	335.858 ± 152.445	33.037 ± 17.994
4.4.	2945.256 ± 306.004	218.501 ± 26.569	1773.583 ± 490.115	6.711 ±1.204	117.325 ± 37.947	237.891 ± 87.707	26.615 ± 7.777
4.5.	4847.427 ± 881.220	215.933 ± 30.091	6371.991 ± 1756.093	13.803 ± 3.719	178.533 ± 16.093	681.203 ± 172.902	56.608 ± 12.201
DTV	36	85	140	35	100	-	-
GTV	60	100	200	50	100	20	-

*Abbreviations.* LCu, copper levels in aquatic sediments; LPb, lead levels in aquatic sediments; LZn, zinc levels in aquatic sediments; LNi, nickel levels in aquatic sediments; LCr, chromium levels in aquatic sediments; LAs, arsenic levels in aquatic sediments; LFe, iron levels in aquatic sediments; 1.1., site S1 (July 2011); 1.2., site S2 (July 2011); 1.3., site S3 (July 2011); 1.4., site S4 (July 2011); 1.5., site S5 (July 2011); 2.1., site S1 (November 2011); 2.2., site S2 (November 2011); 2.3., site S3 (November 2011); 2.4., site S4 (November 2011); 2.5., site S5 (November 2011); 3.1., site S1 (March 2012); 3.2., site S2 (March 2012); 3.3., site S3 (March 2012); 3.4., site S4 (March 2012); 3.5., site S5 (March 2012); 4.1., site S1 (July 2012); 4.2., site S2 (July 2012); 4.3., site S3 (July 2012); 4.4., site S4 (July 2012); 4.5., site S5 (July 2012); DTV, Dutch targer values for TE concentrations in aquatic sediments; GTV, German target values for TE concentrations in aquatic sediments.

Mineral deposits from the Bor metallogenic zone are known to contain elevated levels of Cu, Zn, Pb and As [42,43]. The processing of such ores is therefore expected to result in extensive deposition and accumulation of these TEs in aquatic sediments. Our results support this hypothesis. Interestingly, in three out of four seasons the highest LCu, LPb, LZn and LAs were found at site SR. Given the values of physico-chemical properties of water, this seems paradoxical because this site provides the most suitable conditions for aquatic life among investigated sites. TE loadings in sediments accurately reflect pollution history [44], but, as shown in the present study, they may fail to disclose the recent status of aquatic pollution. One plausible explanation for such high TE accumulation in aquatic sediments may be related to the disaffected Roman mines from the Ravna River Basin and the upper course of the Pek River, such as those uncovered at Donja Bela Reka [45].

Among TEs, very high LAs in aquatic sediments are of particular interest because this element is very toxic to living organisms [7]. The background As levels in soil range from 1 to 40 mg kg^-1^ d.w., with average values around 5 mg kg^-1^ d.w. [46]. At all sites, LAs greatly exceeded this range, whereas the median level was 50 times higher than the aforementioned value. The outdated technology used for copper production (viz. pyrometallurgy) is regarded as the main source of As pollution in the Bor area [40]. The major problem related to arsenic toxicity is the inorganic As, which is considered 500 times more harmful than the organic form [46]. The Copper Smelter Complex Bor (RTB Bor) alone is estimated to release about 300 t of inorganic As per year [42]. These data highlight the potential risks that non-ferrous metal mining, smelting and processing operations located in the Bor area may pose to aquatic ecosystems within the lower Danube Basin.

Future studies should comprehensively examine the metal bioavailability in aquatic sediments, because this factor is essential for interactions between the abiotic and biotic components of an aquatic ecosystem [2]. A frequent approach assesses metal bioavailability based on the acid volatile sulfides (AVS) and its ratio to simultaneously extracted metals (SEM). The difference of SEM to AVS shows if sufficient sulfides are present in aquatic sediments in order to immobilize and precipitate divalent metals in the form of insoluble sulfides. This way, an excess AVS would reduce metal bioavailability [47]. This method is proposed to be used in our future studies.

### Enzymatic activity of sediment bacterial communities

The values for ADA, PDA, UA, PA and CA are provided in Table 4 (as median values with standard deviation). It was found that all enzymatic variables showed equal variance, i.e., homoscedasticity (p > 0.05). Site location had no significant effect on UA and PA (p > 0.05), but significantly influenced ADA, PDA, and CA (p <0.05). Although CA, ADA and PDA correlated significantly with each other, no significant relationships were reported among the other enzymatic variables (Table 2).

**Table 4:** Enzymatic activities of sediment bacterial biota

	**ADA**	**PDA**	**UA**	**PA**	**CA**
1.1.	0.088 ± 0.013	0.175 ± 0.024	1.281 ± 0.124	49.753 ± 2.056	10.254 ± 2.339
1.2.	6.522 ± 0.528	36.123 ± 2.996	0.732 ± 0.146	44.534 ± 1.383	14.675 ± 1.983
1.3.	0.786 ± 0.0.054	3.027 ± 0.372	0.686 ± 0.095	47.758 ± 5.096	16.123 ± 0.995
1.4.	15.532 ± 1.076	32.524 ± 4.729	0.731 ± 0.063	43.509 ± 2.117	16.296 ± 3.526
1.5.	1.663 ± 0.094	28.125 ± 2.406	0.572 ± 0.858	37.757 ± 3.408	21.615 ± 4.072
2.1.	0.157 ± 0.043	0.467 ± 0.042	1.196 ± 0.132	61.952 ± 5.097	8.757 ± 1.025
2.2.	0.013 ±0.002	0.551 ± 0.037	1.377 ±0.241	63.511 ± 8.341	15.625 ± 2.137
2.3.	1.638 ± 0.274	2.625 ± 0.296	0.224 ± 0.017	59.753 ± 7.524	14.767 ± 2.084
2.4.	9.529 ± 0.739	22.253 ± 4.231	0.799 ± 0.031	70.758 ± 2.306	16.873 ± 2.096
2.5.	8.125 ± 0.665	42.232 ± 3.936	0.318 ± 0.026	64.875 ± 2.114	15.256 ± 1.897
3.1.	0.238 ± 0.026	2.225 ± 0.245	1.420 ± 0.184	41.529 ± 5.051	7.861 ± 1.991
3.2.	0.209 ± 0.143	0.325 ± 0.038	1.444 ± 0.142	60.541 ± 1.303	14.042 ± 3,482
3.3.	0.125 ± 0.024	0.148 ± 0.184	1.403 ± 0.293	62.875 ± 8.967	12.419 ± 3.074
3.4.	5.411 ± 0.793	25.254 ± 3.846	1.658 ± 0.725	58.375 ± 3.006	19.266 ± 0.950
3.5.	2.375 ± 0.196	14.375 ± 1.497	3.151 ± 0.407	61.231 ± 4.942	11.475 ± 3.041
4.1.	0.338 ± 0.026	0.338 ± 0.036	0.282 ± 0.045	15.259 ± 2.098	10.648 ± 2.538
4.2.	0.763 ± 0.065	0.329 ± 0.019	0.275 ± 0.058	13.011 ± 1.795	23.127 ± 3.004
4.3.	0.156 ± 0.016	0.220 ± 0.021	0.277 ± 0.013	9.352 ± 3.128	11.915 ± 2.041
4.4.	37.733 ± 0.329	2.663 ± 0.098	0.526 ± 0.071	19.253 ± 0.967	17.858 ± 3.038
4.5.	12.256 ± 1.285	13.375 ± 2.912	1.662 ± 0.217	12.759 ± 2.445	14.450 ± 1.093

*Abbreviations.* ADA, actual dehydrogenase activity in sediment bacterial communities; PDA, potential dehydrogenase activity in sediment bacterial communities; UA, urease activity in sediment bacterial communities; PA, phosphatase activity in sediment bacterial communities; CA, catalase activity in sediment bacterial communities; 1.1., site S1 (July 2011); 1.2., site S2 (July 2011); 1.3., site S3 (July 2011); 1.4., site S4 (July 2011); 1.5., site S5 (July 2011); 2.1., site S1 (November 2011); 2.2., site S2 (November 2011); 2.3., site S3 (November 2011); 2.4., site S4 (November 2011); 2.5., site S5 (November 2011); 3.1., site S1 (March 2012); 3.2., site S2 (March 2012); 3.3., site S3 (March 2012); 3.4., site S4 (March 2012); 3.5., site S5 (March 2012); 4.1., site S1 (July 2012); 4.2., site S2 (July 2012); 4.3., site S3 (July 2012); 4.4., site S4 (July 2012); 4.5., site S5 (July 2012).

The highest ADA and PDA levels were found for sites S4 and SR, whereas the measured values at sites S1 and S3 frequently varied within the same range. Among investigated locations, site S2 revealed the greatest variation of these enzymatic activities. Because dehydrogenase activity is sensitive to heavy metal pollution [15,19], such fluctuations of ADA and PDA in aquatic sediments may suggest that this site was exposed to different levels of anthropic pollution throughout the experimental period.

Depending on season, UA level displayed a heterogeneous pattern of spatial variation, but the measured values regularly lied within a small range. Therefore, UA in aquatic sediments appear to be less sensitive to heavy metal pollution than are ADA and PDA. The highest measured values were reported in March 2012, regardless of location. Similar results were obtained in a comparative investigation of soil UA in spring and summer [48]. Negative correlation between sediment/soil temperature and UA level may help explain these results [49].

Although PA exhibited an erratic seasonal variation among sampling sites, the measured values lied within a relatively small range. This, along with the results of previous investigations [15,18], suggested that PA is likely to be less effective than dehydrogenase activity (i.e., either ADA or PDA) in detecting inorganic pollution in aquatic sediments. CA varied among different sites and season, but its levels were systematically shown to be minimal at site S1. Positive association between pH and CA, as shown in Table 2, may account for the observed pattern of spatial variation for this enzymatic activity.

These results clearly show how difficult it is to classify polluted sites based on assessment of single enzymatic criteria, which reveal such high variations depending on season and location. Consequently, an alternative approach is required to achieve reliable results when using microbial enzymatic activity for biomonitoring pollution status in aquatic sediments.

### Interactions between abiotic and enzymatic variables

The overall canonical correlation was substantial and highly significant (canonical R = 0.98, p < 0.01). The first canonical root extracted 65.508% of the variance of abiotic variables, and 100.00% of the variance of enzymatic variables. Given the abiotic variables, only 13.508% of the variance in enzymatic activities of sediment bacterial biota can be explained based on the first canonical root. This suggested a relative lack of correlations between these two sets of variables. Table 5 gives the canonical loadings for both sets of variables. The highest loadings among abiotic variables were observed to occur for WEC and pH; therefore, one can conclude that these physico-chemical properties of water accounted for most of the significant canonical correlation between the two sets of variables.

**Table 5:** Canonical loadings for abiotic and enzymatic variables

**Abiotic variables**		**Enzymatic variables**	
DO	-0.314	ADA	-0.342
pH	-0.722	PDA	-0.444
WEC	0.615	UA	-0.295
LCu	-0.207	CA	0.314
LPb	0.174	PA	-0.641
LZn	-0.321		
LNi	-0.078		
LCr	-0.273		
LAs	-0.179		
LFe	0.253		

*Abbreviations.* DO, dissolved oxygen in water; pH, activity of the (solvated) hydrogen ions in water; WEC, electrical conductivity of water; LCu, copper levels in aquatic sediments; LPb, lead levels in aquatic sediments; LZn, zinc levels in aquatic sediments; LNi, nickel levels in aquatic sediments; LCr, chromium levels in aquatic sediments; LAs, arsenic levels in aquatic sediments; LFe, iron levels in aquatic sediments; ADA, actual dehydrogenase activity in sediment bacterial communities; PDA, potential dehydrogenase activity in sediment bacterial communities; UA, urease activity in sediment bacterial communities; PA, phosphatase activity in sediment bacterial communities; CA, catalase activity in sediment bacterial communities.

Investigating the response of soil biological activities to soil amendment with Zn, Pb and Cd chlorides, Zn was found to have the highest inhibitory on soil dehydrogenase, acid and alkaline phosphatase, arylsulfatase, urease, and nitrification potential [50]. These findings may explain our results, i.e., among TEs, LZn exerted the strongest influence on bacterial enzymatic activity (Table 5). Although ADA and CA were significantly correlated with pH, and PA displayed a moderate relationship with LFe, the enzymatic activities were generally independent of abiotic variables (Table 2). Recently, ADA, UA, PDA, CA and PA levels in soil were shown to be inhibited in a dose-dependent manner under laboratory conditions by either Zn, Cd or Pb [15]. However, this linear relationship between microbial enzymatic activities and TE levels in soil/sediments may be difficult to extrapolate to field surveys. The present study showed that inorganic pollution in investigated area affected CA, ADA, and PDA, but much less so UA and PA (Table 5). Such discrepant results were attributed to complex intercorrelations among TEs in sediments and physico-chemical properties of water (as shown in Table 2); and differences in habitat conditions and population structure of sediment bacterial biota. This suggested that in natural environments sediments are key catalysts of water quality and function as heterogeneous systems, wherein various enzymatic activities complexly interact to induce biochemical transformation of anthropic contaminants.

### EISQ as bioindicator of environmental pollution in aquatic sediments

Most studies used EISQ either to assess the separate effects of short-term single exposure to heavy metals under laboratory conditions [15,19], or employed this bioindicator in non-repeated field surveys [20]; therefore, such results may reflect a particular situation at a specific moment in time. The novelty of our study is the systematic approach to EISQ validity as a bioindicator of environmental pollution by performing repeated measurements of this parameter in the same places at different moments in time.

**Figure 2:**
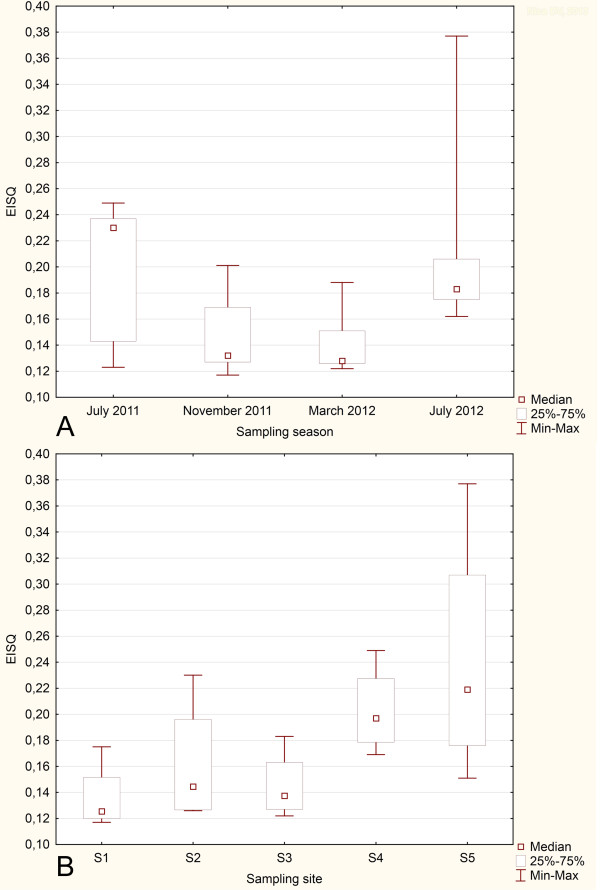
A) EISQ variation at the investigated sites. B) EISQ variation depending on sampling site location.

Our results showed that EISQ values were homoscedastic (p > 0.05), but the measured levels were not significantly different among sampling locations (p > 0.05). The median values were lower in autumn and winter than in summer (Figure 2A); this may be related to the reduced activity of microbial population due to low temperature during autumn and winter [51].The lowest EISQs were found at sites S1, S2 and S3 (Figure 2B), suggesting that the native bacterial communities were more affected by anthropic pollution than those inhabiting sites S4 and SR. Site location may help explain these results (Figure 1). Thus, sites S1 and S2 were placed next to major pollution sources: site S1 in the ”Jama Bor” underground mine; site S2 near the Cerovo open pit mine; site S3 downstream from the junction of two heavily polluted water streams, i.e., the Kriveljska and Bor rivers. The reference site (SR) displayed the highest EISQ variability, but the corresponding median indicated that this habitat was less damaged by inorganic pollution than any other site (Figure 2B). Although EISQ medians varied narrowly, regardless of season (Figure 2A), our results clearly showed that this biomarker is sensitive enough to detect even low spatial variations in overall enzymatic activity of sediment bacterial communities (Figure 2B).

WEC correlated negatively with EISQ (r_s_ = -0.47, p < 0.05), but it was generally independent of abiotic variables (-0.16 < r_s_ < 0.35, p > 0.05). Minimally impacted streams were demonstrated to have lower WEC than highly impacted streams [52]. Therefore, one can expect that EISQ is reversely linked to the degree to which aquatic habitats have been affected by anthropic pollution. Given the lack of correlation between TE concentrations in sediments and EISQ, the total enzymatic activity of sediment bacteria appear to be associated with the current status of aquatic pollution rather than being related to the duration of exposure to inorganic pollutants from aquatic sediments. Such findings may be associated with enhanced bacterial tolerance as a result of long-term exposure to elevated levels of TEs; and with reduction of TE toxicity over time due to geochemical and bacterial transformation [53]. Organic pollutants can also induce EISQ variation in aquatic sediments [20]. However, in the Bor area, this enzymatic bioindicator is mainly associated with inorganic pollution since such compounds are known to regulate the physico-chemical properties of water streams in this region [30,32].

**Figure 3:**
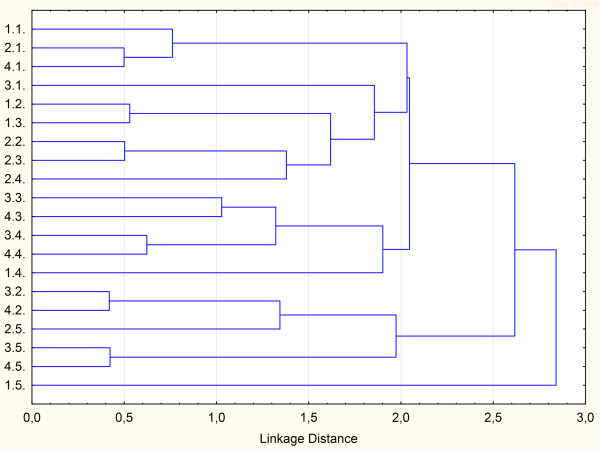
**Dendrogram of the CA based on physico-chemical properties of water, trace element loadings, and total enzymatic activity in aquatic sediments.***Legend.* 1.1., site S1 (July 2011); 1.2., site S2 (July 2011); 1.3., site S3 (July 2011); 1.4., site S4 (July 2011); 1.5., site S5 (July 2011); 2.1., site S1 (November 2011); 2.2., site S2 (November 2011); 2.3., site S3 (November 2011); 2.4., site S4 (November 2011); 2.5., site S5 (November 2011); 3.1., site S1 (March 2012); 3.2., site S2 (March 2012); 3.3., site S3 (March 2012); 3.4., site S4 (March 2012); 3.5., site S5 (March 2012); 4.1., site S1 (July 2012); 4.2., site S2 (July 2012); 4.3., site S3 (July 2012); 4.4., site S4 (July 2012); 4.5., site S5 (July 2012).

The constrained Ward’s method for cluster analysis enables the resulting groups to be joined such that increase in within-group variance is minimal [54]. The corresponding dendrogram is shown in Figure 3. Site S1 corresponded to the most impacted stream, which provides the most extreme habitat conditions for sediment bacterial communities due to low pH and DO, but high WEC levels. The most suitable habitat conditions were reported for the reference site (SR), although this site displayed, on average, the highest TE concentrations in sediments among investigated sites. Site S4 proved to be slightly affected by anthropic pollution than site S3, irrespective of season. Different water discharges and degrees of inorganic contamination may induce in these two locations different responses of sediment bacterial communities to inorganic pollutants. Site S2 revealed the most heterogeneous grouping, with the highest impact of anthropic pollution being observed during March 2012 and July 2012. This may be related to the reopening of the Cerovo open pit mine in 2012 [55]. These results therefore suggested that sites with similar pollution patterns can be appropriately classified by applying cluster analysis to physico-chemical properties of water, TE loadings and total enzymatic activity in aquatic sediments. This statistical approach has a great cumulative power, which provides an integrated overview to environmental researchers if they want to simultaneously identify the impact of inorganic pollution at different trophic levels.

## Conclusions

The environmental impact of inorganic pollution is pronounced in water streams adjacent to

Copper Smelter Complex Bor (RTB Bor, Serbia), with Cu, Zn, Pb, and As being the main determinants of aquatic pollution pattern. Depending on season and site location, catalase activity (CA), potential dehydrogenase activity (PDA), actual dehydrogenase activity (ADA), urease activity (UA), and phosphatase activity (UA) in aquatic sediments displayed different responses to inorganic pollution. A new approach is therefore employed to overcome this impediment by cumulatively assessing the impact of inorganic pollution on these enzymatic activities in bacterial biota. It is shown that the median values of the enzymatic indicator of soil quality (EISQ) correlate negatively with water electrical conductivity, but not with the levels to which trace elements accumulated in aquatic sediments. Moreover, this bioindicator appears to be reversely associated with the distance from the major sources of pollution. In sum, EISQ shows a great potential for assessing the current status of inorganic pollution in aquatic environments.

## Experimental

### Sampling sites

Five tapping points have been chosen to collect water and bedded sediment samples in water streams around the city of Bor (Serbia). All sampling sites were located within a 15 km- radius of major sources of pollution, such as Bor, Cerovo, or Krivelj open pits (Figure 1). The first sampling point (S1; location: 44.5368° lat. N, 22.5403° long. E) lies 2.4 km northwest of the city of Bor, in the “Jama Bor” underground mine. A suburban settlement of Bor city is located only 200 m far away from this site, near the wastewater stream originating from the underground mine. The second sampling point (S2; location: 44.1018° lat. N, 22.1548 long. E) is a tributary of the Cerovo River, located northwest, about 12.10 km far away from the city of Bor, and 2.5 km far away from the village of Mali Krivelj. The third sampling point (S3; location: 44.1512° lat. N, 22.1238° long. E) and the fourth sampling point (S4; location: 44.1466° lat. N, 22.1229° long. E) are located near the junction of Bor and Veliki Krivelj rivers: S3 100 m downstream, and S4 50 m upstream, respectively. The river mouth lies about 10.10 km southeast of the city of Bor, and only 3.9 km far away from the village of Slatina. The fifth sampling site (SR; location: 44.4593° lat. N, 22.1385° long. E) is the Ravna Reka River, 9.65 km east of the city of Bor city, and about 2.03 km east of the village of Donja Bela Reka.

Site SR was considered as the reference site because, to our knowledge, the Ravna river Basin was not severely affected by anthropic pollution during the past decades or so [45]. Site S1 was regarded as the most polluted location due to its close location to “Jama Bor” underground mine [26]. Sites S2 was thought to be less polluted than site S1 because the Cerovo open pit mine was reopened only in May 2012 [54]. Despite their close location, sites S3 and S4 were expected to show different levels of pollution as result of different water discharge [26].

### Physico-chemical properties of water and TE concentrations in sediments

The physico-chemical parameters of water were measured by using a handheld multimeter WTW pH 340i/SET fitted with specific sensors for each parameter. These measurements were performed on-the-spot by the same researcher for all the investigated sites. Because the physico-chemical properties of water reflect the status of water quality [33,34], these criteria were used as screening benchmarks for selecting the reference site.

For each site, aquatic sediments were sampled in triplicate from the top layer of the bedded sediments. Samples were collected in sterile bottles to prevent their contamination. During the next day, the deposit was filtered through ash-free filter paper after the sediments settled to the bottom of sampling bottles. For each sample, the total amount of sediment was split in two batches: the first batch was used to measure TE concentrations in sediments, whereas the second batch was used to assess the enzymatic activity in sediment bacterial biota. The corresponding samples were oven dried at 105°C to constant weight, and then weighed by using an analytical balance to the nearest 0.01 mg. The weighing procedure was repeated five times and only the mean value was taken into account.

The sediment samples were calcinated in a muffle furnace, wherein the temperature was gradually increased up to 550°C and maintained constant for 4 hours. The ash was dissolved in 20 mL of 0.5 N HNO_3_ solution and filtered through ash-free filter paper before analysis [56]. For each sample, the volume was brought to 50 mL with 30 mL of 0.5 N HNO_3_ solution. The nitric acid (65%, ρ = 1.39 g/cm^3^) used to prepare digestion solution (HNO_3_ 0.5 N) was purchased from Sigma-Aldrich Chemie GmbH (Buchs, Switzerland) and was trace metal grade (Suprapur).

To assess Cu, Pb, Zn, Ni, Cr, As, and Fe concentrations in the filtrate we used flame atomic absorption spectrophotometry with an acetylene-nitrous oxide flame (Perkin-Elmer 403 AAS). For each analyzed TE, stock solutions (1000 ± 5 mg kg^-1^ d.w.) were purchased from May and Baker Group and prepared in three different concentrations for constructing the corresponding calibration curves. All glassware was treated with Pierce solution 20% (v/v), rinsed with cold tap water, treated with 20% (v/v) nitric acid, and then rinsed again with bidistilled water. All blanks and duplicate samples were analyzed during the procedure. NCS Certified Reference Material-DC 85104a and 85105a (China National Analysis Center for Iron&Steel) was separately analyzed for quality assurance. Percentage recoveries for TE analysis varied between 85% and 105%. TE levels in sediments were expressed as milligram per kilogram dry weight (mg kg^-1^ d.w.). All the measurements were performed by the same researcher in the same conditions for all sampling sites and seasons.

### Sediment bacteria enzymatic activity

Fresh sediments were used to assess the influence of inorganic pollution on enzymatic activity of microbial communities within the area of interest. The samples were weighed as described above.

To determine catalase activity (CA), for each sample three grams of sediment were transferred in sterile polyethylene tubes, together with 10 mL phosphate buffer and 2 mL of 3% solution H_2_O_2_. Next, they were incubated at room temperature (t = 22°C) for 1 h. The reaction was stopped by adding 10 mL of 4 N H_2_SO_4_ solution and 78 mL bidistilled water. The samples were then filtered through ash-free filter paper, and 25 mL filtrate/sample were put in an Erlenmeyer glass flask. After that, 2.5 mL of 4 N H_2_SO_4_ solution were added in each Erlenmeyer glass flask. The mixture was titrated with a 0.05 N KMnO_4_ solution. CA was finally expressed as milligrams H_2_O_2_ per gram of sediment [57].

Urease activitity (UA) assesses the rate of urea decomposition in ammonia (NH_3_) and carbon dioxide (CO_2_). For each sample, five grams of sediment were put in a sterile polyethylene tube containing 2 mL toluene, 5 mL phosphate buffer, and 5 mL of 5% urea solution (CH_4_ON_2_). The mixture was incubated at 37°C, for 24 h. The ammonia was extracted with a 2 N potassium chloride (KCl) solution, and determined by spectrophotometric nesslerization at 445 nm; the corresponding calibration curve was created using an ammonium chloride (NH_4_SO_4_) solution [56]. UA was expressed as miligrams ammonium ions (NH_4_^+^) per 100 grams of sediment.

Phosphatase activity (PA) was estimated based on hydrolytic separation of phenyl phosphate by phosphomonoesterases; the final products are disodic phosphate and phenol. The latter compound reacts with Gibbs reactive (2,6-dibromchinon-chloramide) resulting in a blue precipitate. For each sample, about 2.5 g sediment was put into a test tube containing 10 mL of 0.5% disodic phosphate solution. The mixture was incubated at 37°C for 48 h. Next, 50 mL of ammonium aluminium sulfate, i.e. NH_4_Al(SO_4_)_2_×12H_2_O, were added to each test tube and the mixture was then filtered through ash-free filter paper. From each test tube, 1 mL filtrate was transferred to an empty test tube, together with 5 mL Borax solution (Na_2_B_4_O_7_ × 10 H_2_0, pH = 9.4). The mixture was brought to a volume of 25 mL with bidistilled water. PA was determined at 597 nm. The calibration curve was constructed by using a 50 μg/mL phenol (C_6_H_5_OH) solution. PA was defined as milligrams phenol per 100 grams of sediment [58].

Actual dehydrogenase activity (ADA) and potential dehydrogenase activity (PDA) were determined using colorimetric measurement of 2, 3, 5-triphenyltetrazolium chloride (TTC) reduction to triphenyltetrazolium formazan [59]. The reaction mixture used for determining ADA contained 3 g sediment, 0.5 mL of 3% TTC solution, and 1 mL bidistilled water. In addition, 1 mL of 3% glucose solution was added to prepare the solution required for assessing PDA values. In both cases, the samples were incubated at 37°C, for 48 h. ADA and ADP were detected by measuring absorbance at 485 nm. The calibration curve was obtained with an etalon solution containing 13.44 mg formazan per 50 mL solution. The measured values of ADA were assessed as miligrams formazan per 10 grams of sediment.

All measurements of enzymatic activity were performed by using a T90 UV/Vis spectrophotometer (PG Instruments, England). For each sampling season, the analytic protocol was carried out in triplicate, in a controlled laboratory environment, by the same researcher during the same day. Finally, the enzymatic indicator of soil/sediment quality (EISQ) was calculated according to the formula:

$$\mathrm{EISQ}=1/\mathrm{nx}\Sigma {\;V}_{r}/V_{\max}$$

where n defines the number of investigated enzymatic activities, V_r_ the value of each enzymatic activity and V_max_ maximal theoretical value of each enzymatic activity [15,17], i.e., the maximum amount of reaction products obtained from the complete degradation of the corresponding enzymatic substrate.

### Statistical analysis

Non-parametric statistic was used for all variables to test for homoscedasticity and significant differences among investigated locations (Levene’s test, df = 5,15; Kruskal-Wallis test, df = 5,15). Canonical correlation analysis (df =50) and Spearman’s rank correlations (15×60 matrix, df = 58) were conducted for assessing relationships among abiotic and enzymatic variables. Finally, we employed single linkage cluster analysis using the constrained Ward’s method (df = 899) to classify the investigated sites depending on physico-chemical properties of water, TE loadings and total enzymatic activity in aquatic sediments. Statistical analyses were performed by using Statistica 10 and Past software package [60,61]. All data are presented as ($\bar{X}$ ± SD). A p value < 0.05 was considered significant.

## Abbreviations

TE: Trace element; ADA: Actual dehydrogenase activity in sediment bacterial communities; PDA: Potential dehydrogenase activity in sediment bacterial communities; UA: Urease activity in sediment bacterial communities; PA: Phosphatase activity in sediment bacterial communities; CA: Catalase activity in sediment bacterial communities; DO: Dissolved oxygen in water; pH: Activity of the (solvated) hydrogen ions in water; WEC: Electrical conductivity of water; LCu: Copper levels in aquatic sediments; LPb: Lead levels in aquatic sediments; LZn: Zinc levels in aquatic sediments; LNi: Nickel levels in aquatic sediments; LCr: Chromium levels in aquatic sediments; LAs: Arsenic levels in aquatic sediments; LFe: Iron levels in aquatic sediments; EISQ: Enzymatic indicator of sediment quality.

## Competing interests

The authors declare that they have no competing interests.

## Authors’ contributions

MNF, VO, RP have contributed mainly to the study design, sample collection, biological and chemical analyses, data interpretation and manuscript preparation of paper. All authors read and approved the final manuscript.
